# Ising Model: Recent Developments and Exotic Applications

**DOI:** 10.3390/e24121834

**Published:** 2022-12-15

**Authors:** Adam Lipowski

**Affiliations:** Faculty of Physics, Adam Mickiewicz University in Poznań, 61-614 Poznań, Poland; lipowski@amu.edu.pl

Solving in his PhD thesis the one-dimensional version of a certain lattice model of ferromagnetism formulated by his supervisor Lenz [1], Ising came to the conclusion that the model fails to describe finite-temperature ferromagnetism and does not seem to be particularly important [2]. Although Ising solution was correct, his predictions on the importance of this model turned out to be grossly inadequate. Indeed, for more than a century, the Ising model, as it is now called, has provided profound insight into the behaviour of a vast variety of interacting many-body systems [3], even outside the realm of physics [4].

The present Special Issue, “Ising Model: Recent Developments and Exotic Applications”, consists of eight original research papers that contribute greatly to our understanding of the Ising model and suggest its possible new applications.

Early research on the Ising model was restricted mainly to ferromagnetic interactions and regular lattices. Gradually, however, interest shifted towards more complex models that incorporate, for example, competing interactions. Recently, there has been a growing interest in studying Ising models of heterogeneous lattices such as random graphs or scale-free networks. In the article [5] published in this Special Issue, Krasnytska et al. examine a model of heterogeneous lattices with an additional disorder related to the strength of the spins. They show that the interplay of lattice and spin strength disorders can result in a new critical behaviour. Their analysis is as an interesting extension of some earlier work based on annealed network approximations [6].

The Ising model, or its more general version—the Potts model—can be used to examine the anomalies of the IT network infrastructure, as demonstrated in the paper by Paszkiewicz [7] that also belongs to the present Special Issue. Such an approach allows one to describe the influence of various disturbances in a network such as, for example, a sudden change in people’s opinion (e.g., due to an election spot), an occurrence of malware in the IT system, or congestion in a computer network. In the era of the Internet of Things or even of Everything, this kind of modelling is likely to play an increasing role.

The next paper in our Special Issue by Valle et al. [8] demonstrates the applicability of the Ising model in econophysics. In particular, they analyse the volatility of returns and argue that this volatility can be modeled with a certain Ising model. Using the Maximum Entropy Principle and machine learning, they infer the coefficients of interactions between assets and analyse to what extent a model with pairwise interactions can explain the behaviour of financial markets. Their work even indicates that during financial turmoil, factors that are external to the financial system make predictions of financial risk much more difficult.

A number of NP-optimization problems can be translated into energy minimization problems of a certain Ising model [9]. Such an approach was used to solve certain polyomino puzzles and is described in the paper by Takabatake et al. [10] in our Special Issue. Their method uses a novel representation of the problem and is computationally less demanding than some previous approaches. They minimize the energy using Hopfield neural networks and demonstrate the effectiveness of their method for some more general polyominoes as well.

In the next paper of our Special Issue, Žukovič et al. argue that the Ising model can be used for the interpolation of spatial data [11]. Such techniques are needed when, for example, some parts of a satellite photo of a certain area are missing due to cloudiness. The authors demonstrate that their approach based on the Ising model offers a very good computational performance, even in comparison o more sophisticated techniques based on kriging or machine learning. Since the amount of data generated by satellites or drones has been rapidly growing recently, it is likely that the demand for such methods will also increase.

The idea that an image can be represented as a certain configuration of the Ising spins appears also in the paper by Choi et al. [12]. They show that a relatively small fraction of spins is sufficient for a reliable restoration of the entire image. In their paper, Choi et al. examine the security risks associated with the reconstruction of certain biometric templates, e.g., a human iris, and argue that their method should be considered as complementary to cancellable biometrics or some schemes of biometric cryptosystem.

Up and down spins in the Ising models can be also interpreted as left and right enantiomers of chiral molecules. Using such an analogy, Dutta and Gellman developed adsorption thermodynamics of chiral molecules [13]. They argue that adsorption from racemic mixtures of enantiomers is directly analogous to the Ising model’s behaviour. An important conclusion of their research, especially in the context of pharmaceutical and biochemical industries, is that enantiomer purification is to some extent similar to phase separation in the Ising model and can be achieved using achiral surfaces.

In yet another paper of the Special Issue, Kryzhanovsky et al. developed an analytical method to examine the Ising model on lattices of large dimensions *d* [14]. Their approach enables them to calculate the critical temperature, free energy, or heat capacity and gives some insight into the behaviour of the density of state. Although the method is less accurate for d<4, the agreement with numerical simulations for d>4 is remarkably satisfactory. 
